# Drivers of Cape Verde archipelagic endemism in keyhole limpets

**DOI:** 10.1038/srep41817

**Published:** 2017-02-02

**Authors:** Regina L. Cunha, Jorge M. Assis, Celine Madeira, Rui Seabra, Fernando P. Lima, Evandro P. Lopes, Suzanne T. Williams, Rita Castilho

**Affiliations:** 1 Centre of Marine Sciences - CCMAR, Universidade do Algarve, Campus de Gambelas, 8005–139 Faro, Portugal; 2 CIBIO, Centro de Investigação em Biodiversidade e Recursos Genéticos, Universidade do Porto, Campus Agrário de Vairão 4485-661 Vairão, Portugal; 3 Universidade de Cabo Verde, Departamento de Engenharias e Ciências do Mar, CP 163, São Vicente, Cabo Verde; 4 Department of Life Sciences, The Natural History Museum, London SW7 5BD, United Kingdom

## Abstract

Oceanic archipelagos are the ideal setting for investigating processes that shape species assemblages. Focusing on keyhole limpets, genera *Fissurella* and *Diodora* from Cape Verde Islands, we used an integrative approach combining molecular phylogenetics with ocean transport simulations to infer species distribution patterns and analyse connectivity. Dispersal simulations, using pelagic larval duration and ocean currents as proxies, showed a reduced level of connectivity despite short distances between some of the islands. It is suggested that dispersal and persistence driven by patterns of oceanic circulation favouring self-recruitment played a primary role in explaining contemporary species distributions. Mitochondrial and nuclear data revealed the existence of eight Cape Verde endemic lineages, seven within *Fissurella*, distributed across the archipelago, and one within *Diodora* restricted to Boavista. The estimated origins for endemic *Fissurella* and *Diodora* were 10.2 and 6.7 MY, respectively. Between 9.5 and 4.5 MY, an intense period of volcanism in Boavista might have affected *Diodora*, preventing its diversification. Having originated earlier, *Fissurella* might have had more opportunities to disperse to other islands and speciate before those events. Bayesian analyses showed increased diversification rates in *Fissurella* possibly promoted by low sea levels during Plio-Pleistocene, which further explain differences in species richness between both genera.

Remote oceanic archipelagos are the ideal setting for studying patterns and processes underlying speciation. While insular terrestrial communities can be formed either by immigration or from a few colonization events and subsequent *in situ* diversification^1^, phylogeographic patterns of marine species inhabiting island settings can be confounded by recurrent episodes of long-distance dispersal. The volcanic origin of oceanic islands and their circumscribed geographic boundaries enable inferring the tempo and sequence of island colonization when geological ages are known^2^.

Isolated islands are expected to have reduced species richness but high levels of endemicity as a result of few colonization events, particularly in older archipelagos^3^. For instance, the reduced dispersal abilities of some Indo-West Pacific turbinid gastropods that lack a long-lived planktonic stage played a crucial role in developing extensive archipelagic differentiation and fine scale endemism^4^. Also, the ongoing emergence and subsidence of islands over geological time is thought to have promoted speciation within this group of marine gastropods at an insular scale^5^.

Allopatric speciation induced by vicariance is sometimes regarded as the main driver of differentiation^6^. Nonetheless, recent developments in methodological approaches to biogeographic analysis^7^, which integrate a wider range of biogeographical processes (i.e. dispersal, extinction, vicariance and duplication)^8^,^9^, showed the importance of dispersal in the diversification processes^10^. For instance, divergence triggered by dispersal events and the organism-specific capacity to occupy suitable habitats and persist, has recently been identified as the main driver of the avian speciation in lowland Neotropical rainforests^11^.

Dispersal in most sessile marine species occurs predominantly during larval stages in which larvae remain in the water column for periods that can vary between days to months before settlement^12^. It is generally assumed that planktotrophic feeding larvae exhibit higher geographic ranges because they are able to remain longer periods in the water column, while lecithotroph yolk-fed larvae complete their metamorphosis without feeding from the plankton and thus are more prone to originate locally structured populations^13^,^14^.

To investigate biogeographical processes and drivers of marine speciation in organisms with pelagic larval development we assessed phylogeographic patterns and inferred dispersal in the keyhole limpets (Vetigastropoda: Fissurellidae) from the Cape Verde Islands. Keyhole limpets of the family Fissurellidae are gastropods that typically have a hole in the top or margin of their limpet-shape shell and live exclusively on rocky substrates^15^. Most of the species feed on algae, sponges or detritus^16^. Fissurelids are gonochoristic (separate sexes), have external fertilization (broadcast spawners) and their breeding season (e.g. within the genus *Fissurella*) occurs predominantly between October and November^17^. A study on larval development of *Fissurella volcano* revealed that the species has short-lived planktonic larvae that remain in the water column up to four days, feeding on yolk reserves before settling^18^,^19^. Larval development of Fissurellidae from the genus *Diodora* may vary from a complete absence of a pelagic larval phase (e.g., *D. graeca*, which has no larval phase^20^) to a planktonic larval period of more than three weeks (e.g., *D. aspera*^21^). The description of Cape Verde fissurellids based on shell characters indicated the existence of twelve *Fissurella* species, seven of them probably endemic, and six *Diodora* species, including only one endemic^22^.

Cape Verde represents an excellent model system for studying speciation and to infer colonization pathways given its remoteness and known geological age of most of the islands. This volcanic oceanic archipelago is approximately 500 km off mainland (Senegal - West Africa) and comprises ten major islands and several islets (Fig. 1). Geochronological data place the age of the islands between 5.9 ± 0.1 million years ago (MYA) and 25.6 ± 1.8 MYA^23^,^24^,^25^. The remoteness of this ancient archipelago has provided the necessary conditions for marine radiations to occur; the most remarkable within the venomous cone snails of the genus *Conus* where more than 60 endemic species described^22^,^26^. This extraordinary diversity was driven by the low dispersal ability of *Conus* non-planktonic lecithotrophic larvae in combination with repeated instances of low sea level that isolated populations and promoted differentiation^27^,^28^.

We modelled dispersal to explain diversification within Fissurellidae lineages from Cape Verde Islands and reconstructed the evolutionary history of this group through time and space. Specifically, our objectives were to: (1) delimit evolutionary lineages within Cape Verde Fissurellidae using mitochondrial cytochrome oxidase subunit I (COI) and nuclear (28S rRNA) sequence data; (2) date major lineage splitting events within the family; (3) analyse heterogeneity in diversification rates through time; (4) estimate ancestral ranges; (5) investigate colonization pathways; (6) quantify the effect of a spatial variable on species richness, and (7) model dispersal using pelagic larval duration (PLD) and prevailing ocean currents as proxies.

## Results

### Phylogenetic reconstruction

Bayesian analysis using the combined data set (145 taxa; COI: 540 bp; 28S rRNA: 826 bp) yielded the topology depicted in Fig. 2. BI analysis retrieved two clades of Cape Verde Fissurellidae. One clade included all specimens from Cape Verde that grouped with the included worldwide members of Fissurellinae. The other clade grouped all Cape Verde samples with western Atlantic and Mediterranean *Diodora*.

### Species delimitation tests

The GMYC species delimitation tests clearly rejected the null model that all specimens belong to a single lineage both with COI (*P* = 3.05 × 10^−8^) and the two-gene analyses (COI+28S; *P* = 0) and identified eight Fissurellidae species in the Cape Verde Islands: seven *Fissurella* and one *Diodora* (Fig. 2). Details on species classification are shown in Table S1 from the Supplementary information. SpedeSTEM also identified the same seven *Fissurella* species from Cape Verde but failed to identify the single *Diodora* species from Cape Verde as distinct from the remaining worldwide *Diodora* species (Fig. 2). Specimens from South Africa and Angola were recovered as new species of uncertain generic status and named as Fissurellidae sp. 1 and Fissurellidae sp. 2, respectively.

### Bayesian Analysis of Macroevolutionary Mixtures (BAMM)

Net diversification rates, as estimated by the BAMM model, accelerated during the Late Oligocene-Early Miocene during the diversification of the clades corresponding to *Diodora, Fissurella* and *Emarginula* (Fig. 3a). Effective sample sizes (ESS) ≫ 200 indicated adequate sampling of the posterior. The 95% HPD (highest posterior density interval) sampled by BAMM after analysing 9,001 posterior samples comprised the eight most probable distinct shift configurations. The single best shift configurations sampled by BAMM suggested an increasing of diversification rates in Cape Verde *Fissurella* (excluding *Fissurella* cf. *salvatiana*), with a probability of *f* = 0.68 (Fig. 3b). The second-best configuration (*f* = 0.21) suggested an acceleration of diversification rates in the node comprising all Fissurellidae, excluding Hemitominae (*Puncturella* sp. and *Cranopsis cucullata*) (Fig. 3c). An additional minor shift (*f* = 0.1) was identified in the clade including all Fissurellidae except Hemitominae and *Emarginula* (Fig. 3d). Overall extinction rates were 0.046 (0.006–0.109) and remained constant through time. Speciation rates were 0.089 (0.058–0.132) and an overall increase towards the present was identified.

### Dating analysis

The origin of the most recent common ancestor (MRCA) of all Cape Verde *Fissurella* was estimated at 10.21 [7.99–12.72] MYA (node A; Fig. 4) but most of the diversification within this group occurred during the Late Pliocene (2.98 [2.19–3.89] MYA; node D, Fig. 4). The stem group age of the single *Diodora* species from Cape Verde (*D. philippiana*) was estimated at 6.74 [5.29–8.41] MYA (node G; Fig. 4).

### Ancestral area estimation

BAYAREALIKE +J was selected as the best-fitting biogeographical model when including all Fissurellidae (Fig. 4). The addition of the “J” parameter for founder-effect significantly increased the likelihood of the traditional model (BAYAREALIKE -ln *L* = 149.74; BAYAREALIKE + J -ln *L* = 119.92; P = 1.1E^−14^, Supplementary information S2A,B). The western Atlantic was the estimated range for the stem lineage of worldwide Fissurellidae (Fig. 4) except for the Hemitominae (*Cranopsis cucullata* and *Puncturella* sp.), which was the Pacific. This model suggests Boavista as the ancestral range of *F.* cf. *salvatiana* and of *D. philippiana*. The inferred ancestral area of the remaining Cape Verde *Fissurella* comprises the northwestern islands of the archipelago (Santo Antão, Ilhéu Raso, Santa Luzia, São Vicente and São Nicolau). Our results suggest a dispersal event towards Sal where *Fissurella* sp. 1 and *Fissurella* sp. 2 originated, and another to Maio where *F. gaillardi* formed. Present-day distribution of *F. bravensis* is Sal, Boavista, Maio, Santiago and São Vicente, which implies dispersal events from the northwestern islands (the ancestral area) towards Sal, Boavista and Maio.

### Shore substrate composition

The total amount per cell of the coastline of rocky substrate (in grey) and sand (in yellow) on each island is shown in Supplementary information S3. The percentage per cell of the coastline of rocky substrate (in grey) and sand (in yellow) on each island is shown in Supplementary information S4. Boavista is the island with the highest percentage of sand whereas Ilhéu Raso and Santiago show the highest percentage of rocky substrate (Supplementary information S4).

### Dispersal potential of keyhole limpets

The simulations using high-resolution ocean current fields over the 10-year period allowed releasing 360 particles per cell (6.39E^6^ passive particles in total). Maximum and average distances, probabilities and drifting time produced by the particles that effectively connected different coastal cells determined for the simulations running 4 and 30 days of passive dispersal are shown in Table 1. The mean distance that particles can reach during four days of passive dispersal is 75.3 ± 75.9 km, which is approximately the same after 30 days (76.1 ± 75.9 km). Mean connectivity probabilities between pairs of islands produced with simulations running 4 and 30 days of passive dispersal are shown in Tables 2 and 3, respectively. Overall probabilities of connectivity between pairs of islands are quite low (between 2.3E^−06^ and 2.2E^−03^). The highest probability of connectivity occurs between São Vicente and Santo Antão and the lowest between Santo Antão and Maio. The degree of connectivity between islands inferred in network analysis running for four or 30 days of passive dispersal are shown in Fig. 5(a and b), respectively. Only stronger links are depicted. Modularity values indicated good divisions >0.3^29^. Lagrangian Particle Simulation performed to estimate the dispersal potential of keyhole limpets through the Cape Verde archipelago is shown in http://rcastilho.pt/R2C2/dispersal_movie.html. The animation shows different source locations releasing particles every 12 hours from July to November 2010. The particles are allowed to drift for a maximum of 30 days with effective dispersal measured when they end up on shore.

## Discussion

We performed a comprehensive sampling through the archipelago (only the two younger islands, Fogo and Brava, have not been sampled) and phylogenetic analyses recovered all sampled individuals in two monophyletic groups (Fig. 2). One clade corresponding to the genus *Fissurella*, grouped with the worldwide Fissurellinae and the other with *Diodora* from the Mediterranean and western Atlantic. Both species delimitation tests (SpedeSTEM and GMYC) indicated the existence of seven Cape Verde endemic *Fissurela* species. SPedeSTEm did not separate Cape Verde specimens of *Diodora* from the remaining *Diodora* species of the Mediterranean, western Atlantic and Pacific, whereas GMYC did recognise Cape Verde specimens as a distinct entity. However, results from both GMYC and genetic distances between Cape Verde *D. philippiana* and its sister group (*D. graeca, D. cayenensis* and *D. listeri*; Fig. 2), strongly suggest that these specimens should be considered an endemic species. These results are not in agreement with a morphology-based study that reported the existence of twelve *Fissurella* species (seven of them probably endemic) and six *Diodora* species (only one endemic) in the Cape Verde Islands^22^. This study was based on shell characters only, which often exhibit high degree of plasticity^30^. The alternative hypothesis that all ten reported non-endemic species (five *Fissurella* and five *Diodora*) could have been overlooked during field sampling seems unlikely.

**Figure 1:**
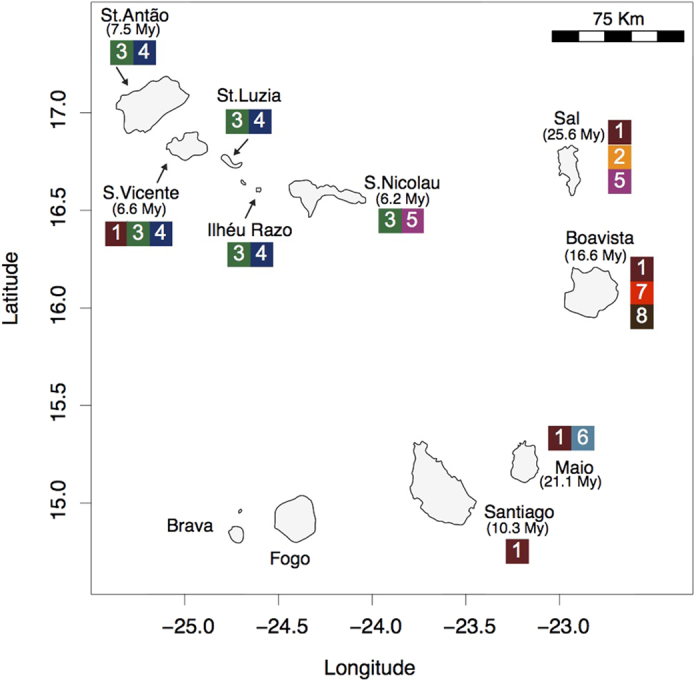
Map of the Cape Verde archipelago. Geological ages of the islands (in million years, when known), latitude and longitudes are shown. Numbers in the squares represent the occurrence of Cape Verde fissurellids (1: *Fissurella bravensis*; 2: *Fissurella* sp. 1; 3: *F. afra*; 4: *F. verna*; 5: *Fissurella* sp. 2; 6: *F. gaillardi*; 7: *Diodora philippiana*; 8: *F.* cf. *salvatiana*). Figure generated using the worldHires (http://CRAN.R-project.org/package=mapdata) function implemented in R language (R Core Team (2015). R: A language and environment for statistical computing. R Foundation for Statistical Computing, Vienna, Austria. URL https://www.R-project.org/) (version 3.3.1), which uses publicly available coastline coordinates from the NOAA National Geophysical Data Center (http://www.ngdc.noaa.gov/mgg/shorelines/shorelines.html) and Adobe Illustrator CS6 (version 16.0.0) (http://www.adobe.com/products/illustrator.html).

**Figure 2:**
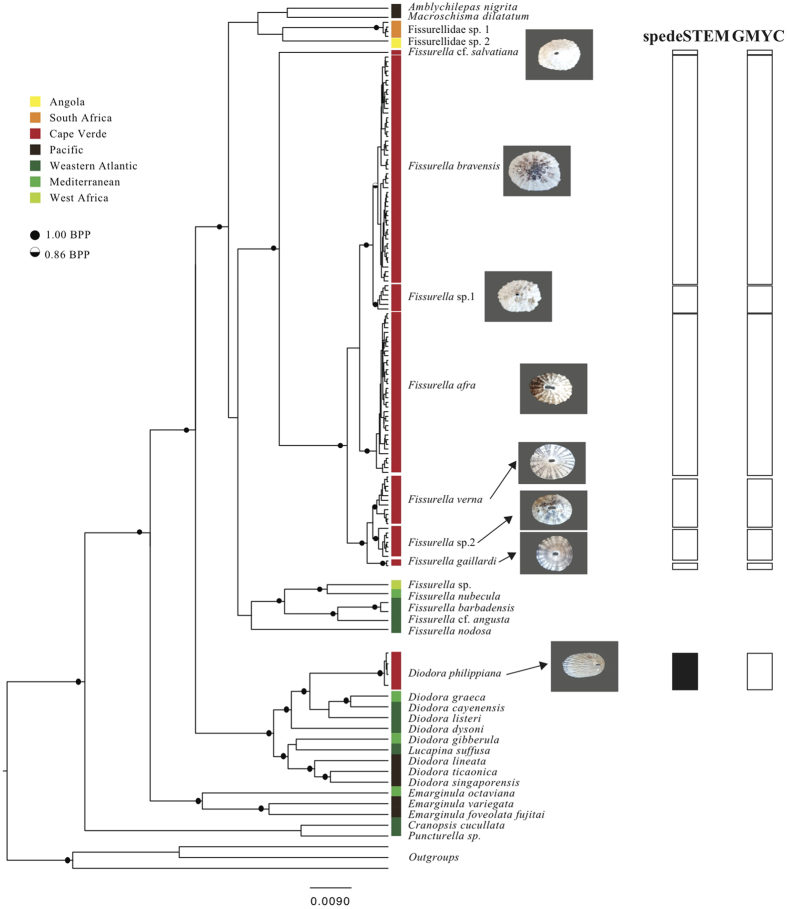
Phylogenetic relationships of Fissurellidae based on a Bayesian inference analysis of mitochondrial COI and nuclear 28S rRNA genes produced by Beast. Bayesian posterior probabilities above 50% are indicated over the branches (full black circles: BPP = 100%; half-black circle: BPP = 86%). Geographical origin of the taxa is depicted in colours. Photos of the shells corresponding to each of the Cape Verde *Fissurella* and *Diodora* species are shown. Assignments of each Cape Verde sample to delineated entities from GMYC and SpedeSTEM species delimitation tests are shown by rectangles. Colour of cluster boxes corresponding to each species indicates mismatch (black) and agreement (white) between both methods. Figure generated in Adobe Illustrator CS6 (version 16.0.0) (http://www.adobe.com/products/illustrator.html) and photos taken by R. L. Cunha.

Our results both regarding the number of Cape Verde Fissurellidae species (Fig. 2) and their distributional ranges (Fig. 1) are somewhat surprising. The level of endemism found in Cape Verde *Fissurella* was unexpected, even for organisms with a theoretical pelagic larval phase of four days, considering that distances between islands are as little as 17 km (e.g., between Ilhéu Raso and S. Nicolau; Fig. 1), and according to our simulations, the mean distance that a particle can reach after four days of passive dispersal is 75.3 ± 75.9 km (Table 1). For instance, the marine snail *Tegula funebralis*, which has a 5-day larval period, shows no genetic structure between Oregon and the Californian coastline^19^, which represents a much larger distance than between most of islands of the Cape Verde archipelago. Nonetheless, this coastal species coexists with its sister species along the continental shore, where no strong physical barriers were identified. It was suggested that transient allopatry determined by historical episodes of climatic fluctuations and shifting currents or ecological barriers could be the main driver of speciation^31^. Considering the extremely low estimated probabilities of connectivity between Cape Verde islands based on four or 30 days of passive dispersal (e.g., Ilhéu Raso *vs*. S. Nicolau, *P* = 1.6E^−04^ for both 4 and 30 days; S. Nicolau *vs*. Ilheu Raso, *P* = 1.8E^−03^ for 4 days or 1.9^−03^ for 30 days; Table 2), diversification of Cape Verde *Fissurella* was most likely promoted by patterns of ocean circulation that favour local retention.

**Figure 3:**
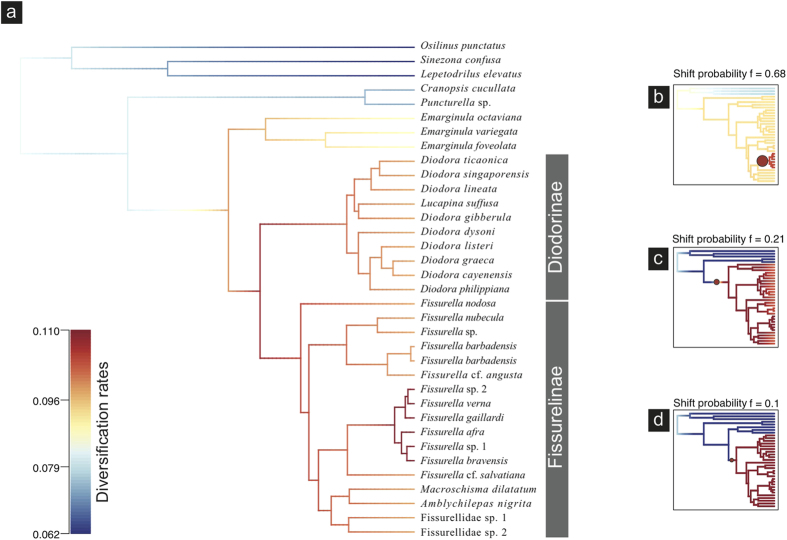
(**a**) BAMM phylorate plot showing the average net diversification rates along each branch of the Fissurellidae. Warmer colours denote faster diversification rates; (**b–d**) the three credible rate shift configurations with the highest posterior probability. Circles denote locations of rate shifts and are proportional to the overall marginal probability of a shift on the branch. One shift with the highest probability (*f* = 0.68) is located at the Cape Verde *Fissurella* clade, excluding *Fissurella* cf. *salvatiana*; the second most-credible shift with probability *f* = 0.21 is located at the node comprising all Fissurellidae, excluding Hemitominae (*Puncturella* sp. and *Cranopsis cucullata*) and an additional minor shift (*f* = 0.1) was identified in the clade including all Fissurellidae except Hemitominae and *Emarginula*.

**Figure 4:**
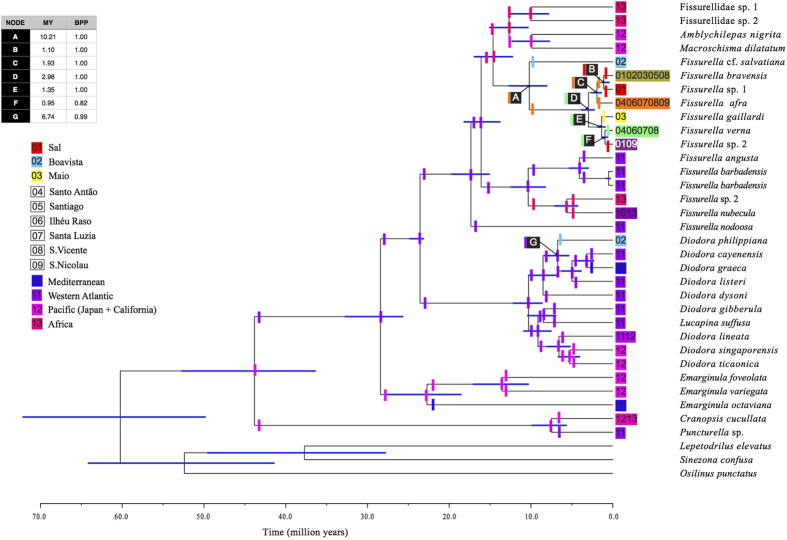
Beast maximum clade credibility chronogram showing main cladogenetic events within Fissurellidae based on mitochondrial COI and nuclear 28S rRNA genes with ancestral estimation inferred with BioGeoBEARS. Age estimates in million years and Bayesian posterior probabilities (BPP) of Cape Verde clades are shown on the table. The corresponding 95% highest posterior density intervals (blue bars) are depicted. Internal coloured vertical bars at branches indicate main ancestral areas recovered by BioGeoBEARS under the selected BAYAREALIKE+J model immediately following a speciation event whereas at nodes indicate ancestral ranges before speciation. Numbers at the coloured squares indicate present-day (tips) distribution of species. In the legend, islands that are not represented, individually, by a single species are not coloured.

BAMM indicated an increase of diversification rates located at the crown age of the clade that included all Cape Verde *Fissurella,* except *F.* cf. *salvatiana* (Fig. 3a and b). Age estimates placed this shift at 2.98 [2.19–3.89] MYA (Fig. 4) at the Plio-Pleistocene boundary. Key innovations or distinct habitat preferences are often invoked to explain higher rates of diversification^32^. We were not able to detect any particular morphological or behavioural feature that could represent an evolutionary advantage for Cape Verde *Fissurella*. All eight species occupied similar habitats and we found no evidence for niche segregation among species (Lopes, Evandro P., pers. obs.).

Another group of marine gastropods with direct development and low dispersal abilities, the cone snails of the genus *Conus* from Cape Verde, also experienced increased speciation rates during the Plio-Pleistocene boundary^28^, which according to the sea level reconstruction of Miller *et al*.^33^ coincided with a ~40 m sea level drop. While Plio-Pleistocene low sea level stands caused important local extinctions on soft-bottom organisms (mostly bivalves) from tropical oceanic islands^34^, sea level fluctuations seemed to have promoted diversification in Cape Verde gastropod species inhabiting hard substrates. The existence of shallow bays and an irregular coastline created additional habitats and opportunities for larval retention during low sea level stands, which might have promoted diversification in both Fissurellidae and *Conus*. No shifts in diversification rates were detected in Cape Verde *Diodora*.

The low levels of connectivity between islands and a shift in diversification rates offer plausible explanations for the level of endemism observed in *Fissurella* but a question still remains: why did *Diodora* not diversify in the Cape Verde archipelago? To address this question, three hypotheses were considered: (i) different sampling efforts for each genus; (ii) differences between the PLD of *Fissurella* and *Diodora*, and (iii) time of origin of each genus in the archipelago.

**Figure 5:**
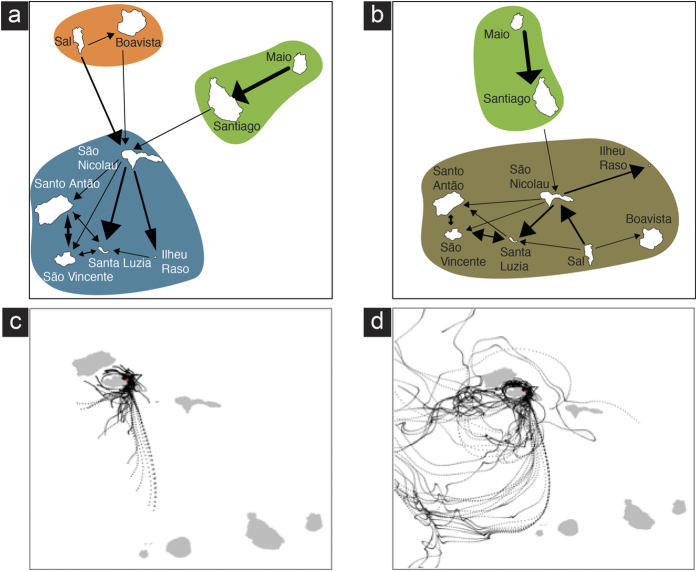
(**a**) Degree of connectivity between Cape Verde Islands inferred in network analysis for the simulation running four days of passive dispersal. Only stronger links are depicted (Modularity: 0.38; three putative oceanographic clusters; significance level of clustering: 0.0048); (**b**) Degree of connectivity between Cape Verde Islands inferred in network analysis for the simulation running 30 days of passive dispersal. Only stronger links are depicted (Modularity: 0.34; two putative oceanographic clusters; significance level of clustering: 0.0291); (**c**) Example of pathways resulting from all particles sent from a coastal cell (red dot) in the simulation running four days of passive dispersal; (**d**) Example of pathways resulting from all particles sent from a coastal cell (red dot) in the simulation running 30 days of passive dispersal. Figures were generated with *igraph* package (version 1.0.1; URL https://cran.r-project.org/web/packages/igraph/index.html) implemented in R language (R Core Team (2015). R: A language and environment for statistical computing. R Foundation for Statistical Computing, Vienna, Austria. URL https://www.R-project.org/) and Adobe Illustrator CS6 (version 16.0.0) (http://www.adobe.com/products/illustrator.html).

The hypothesis that the genus *Diodora* could have been undersampled seems unlikely considering that the sampling effort was equally distributed for both genera. Field work revealed a strong bias in abundances: while more than 300 specimens of *Fissurella* were processed, we were only able to find 26 *Diodora* specimens in total, all from Boavista and all belonging to the same species. The type of larval development of Cape Verde *Diodora* is unknown but under the observed oceanographic conditions, the second hypothesis of a longer PLD to explain the existence of a single species owing to higher connectivity is not supported. Our simulations based on patterns of ocean circulation did not significantly increase effective connectivity between islands even when PLD was increased from four to 30 days of passive dispersal (Tables 1, 2 and 3 and Fig. 5a and b). Note that only effective dispersal events (i.e. those that result in a larva landing on a coast) are reported. Our analyses show very low probabilities of shore-to-shore connectivity (0.003 ± 0.017, on average; Table 1), since most particles are pushed to open waters and only produce effective connectivity events in the very first days of ocean drifting (between 1.29 ± 0.85 and 1.35 ± 1.02 days, on average; Table 1). In fact, only 2.62% of the particles travelled for more than four days, and such an increase in PLD did not expand the maximum travelled distance of the particles (349.3 km; Table 1). As the archipelago is separated from the nearest mainland (the coast of Senegal) by approximately 500 km, this means that island-to-continent dispersal is very unlikely. Finally, the MRCA of *Fissurella* (10.21 [7.99–12.72] MYA) originated three million years earlier than the estimated age for the MRCA of *Diodora* (6.74 [5.29–8.41] MYA) (Fig. 4). Having originated earlier, *Fissurella* might have had more opportunities to disperse to other islands and speciate avoiding the severe volcanic eruptions that struck Boavista between 9.5 and 4.5 MYA^24^, the only island where the *D. philippiana* occurs. This volcanic activity was predominantly distributed along the coastline^24^ and likely had the greatest effect on rocky shore species such as keyhole limpets. Other *Diodora* species also present in Boavista might have become extinct after these volcanic events, before having time to disperse to other islands.

Four of the Cape Verde *Fissurella* lineages are distributed along the coastline of several islands of the archipelago while our sampling suggests that each of the remaining three are restricted to a single island (Fig. 1). Because *Fissurella* keyhole limpets can only be found on rocky substrates, we analysed the effect of hard substrate availability on species richness. The number of *Fissurella* lineages showed, however, no correlation with the total area of rocky coast per island [r(6) = −0.62, p = 0.10] nor with the percentage of rocky coast per island [r(6) = −0.50, p = 0.22, non-significant, see also Supplementary information S3 and S4]. For instance, while Santiago exhibits the largest coastal area with rocky substrate within the archipelago and only one species (*F. bravensis*) can be found, Boavista has three species (*F. bravensis, F.* cf. *salvatiana* and *D. philippiana*) and shows the smallest area of rocky shore.

On the other hand, the degree of connectivity between islands provides the best predictor for present-day distribution of species. A model based on known patterns of ocean circulation and four days of passive dispersal (Fig. 5a) indicated the existence of three main clusters that approximates the present-day distribution of *Fissurella* (Fig. 1). The cluster that includes the northwestern islands (S. Nicolau, Santo Antão, S. Vicente, Santa Luzia and Ilhéu Raso) represents the geographic distribution of *F. afra* and *F. verna*. Present-day distribution of *Fissurella* sp. 2 (Sal and S. Nicolau) is favoured by patterns of ocean circulation that connect these two islands (Fig. 5a). *Fissurella* sp. 1, *F. gaillardi* and *F.* cf. *salvatiana* are restricted to a single island.

The archipelagic endemism in marine sessile organisms reported here was driven by shifts in diversification rates promoted by recurrent episodes of low sea levels during the Plio-Pleistocene boundary and patterns of ocean circulation favouring self-recruitment. The role of dispersal and persistence was determinant in shaping present-day geographic distribution of fissurellid keyhole limpets.

## Methods

### Sampling collection

A total of 363 specimens of Fissurellidae were collected between 2012 and 2013 from 30 locations of the islands of Santiago, Maio, Boavista, Sal, São Nicolau, Santa Luzia, São Vicente and Santo Antão, and Ilhéu Raso of the Cape Verde archipelago (Fig. 1). All specimens were preserved in 96% ethanol. Dr. Rolán, E., a recognized expert in Cape Verde invertebrate fauna and author of^22^, was consulted in the classification of the specimens used in the analyses and their assignment to morphospecies, when possible, otherwise named as *Fissurella* sp. (further details in Table S1 from the Supplementary information).

### DNA extraction and sequencing

Total genomic DNA was isolated from muscle tissue using the cetyltrimethylammonium bromide (CTAB) protocol^35^. A partial fragment of the mitochondrial COI was amplified and sequenced using the universal primers of Folmer^36^ and specific primers designed for Fissurellidae (FissLSU-F- TCCCTCAGTAACGGCGAGTGAAGCG and FissLSU-R - CTTAGCGGATTCCGACTTCCATGGC) amplified a fragment of the 28S rRNA gene. PCR amplifications of the COI and 28S rRNA fragments were carried out in 25 μl reactions containing 5X PCR buffer (Colorless GoTaq® Reaction Buffer, Promega), 0.2 mM of each dNTP, 0.2 μM of each primer, 2 μl of template DNA and GoTaq® DNA polymerase Promega (1 unit) using the following programs: one cycle of 3 min at 95 °C, 40 cycles of 30 s (COI), 45 s (28S rRNA) at 94 °C, 30 s at 50 °C (COI), 45 s at 52 °C (28S rRNA), 2 min at 72 °C, and one cycle of 10 min at 72 °C. PCR amplicons were purified using ethanol/sodium acetate precipitation and directly sequenced with the corresponding PCR primers. Sequencing was performed in an automated sequencer (ABI PRISM 3700) using the BigDye Deoxy Terminator v3.1 Cycle Sequencing Kit (Applied Biosystems), and following the manufacturer’s instructions. All new sequences of Cape Verde Fissurelidae and of specimens from Angola and South Africa were deposited in GenBank (accession numbers in Table S1 from the Supplementary information). All remaining Fissurellidae sequences (one sequence/species) available in GeneBank, sequenced for both COI and 28S rRNA, were used in the phylogenetic analyses (accession numbers in Table S1 from the Supplementary information).

### Sequence Analysis and phylogenetic reconstruction

All DNA sequences were aligned using Mafft version 6.0 (Multiple alignment using Fast Fourier Transform)^37^ using the --auto option that automatically selects the appropriate strategy according to data size. Alignments of both COI (540 bp) and 28S rRNA (826 bp) were unambiguous, and amino acid translations in COI were checked using Mesquite v.3.04^38^.

To analyse phylogenetic patterns within Fissurellidae we performed maximum likelihood (ML) and Bayesian Inference (BI) analyses using PhyML v.3.0^39^ and Beast v.2.3.1^40^, respectively. We used the combined data set (COI: 145 taxa, 540 bp; 28S rRNA: 145 taxa, 826 bp) for both analyses. This data set included all 120 unique COI and 28S haplotypes from Cape Verde samples and the remaining Fissurellidae retrieved from GenBank (accession numbers in Table S1 from the Supplementary information). The Akaike information criterion^41^ implemented in Modeltest v.3.7^42^ selected the TrN + I + G as the evolutionary model that best fits both data sets. The Bayesian analysis was performed using two partitions, COI and 28S rRNA. We used a Yule tree prior^43^ that assumes a constant rate of speciation among lineages and is more appropriate for species-level phylogenies. We used an uncorrelated relaxed, lognormal clock. MCMC analyses were run twice (each run with 100,000,000 generations and a sample frequency of 10,000) following a discarded burn-in of 10,000,000 steps. Length of burn-in was determined by visual examination of traces in Tracer v.1.6^44^. The final tree was produced by TreeAnnotator v.1.8.2^45^ using the “maximum clade creditability” option and mean node height. The convergence to the stationary distribution was confirmed by inspection of the MCMC samples and of effective sample sizes (ESS). ESS values above 200 indicate convergence^46^) in Tracer. BI, biogeographic and dating analyses were performed on the CCMAR Computational Cluster Facility (http://gyra.ualg.pt/) and on the R2C2 research group cluster facility, both at the University of Algarve.

### Species delimitation

For delimiting evolutionary significant units (ESUs) within Fissurellidae we used the method of Pons^47^. This method uses the general mixed Yule-coalescent (GMYC) model to identify “a threshold time before which all nodes reflect diversification events and after which all nodes reflect coalescent events”^47^. We used the single-threshold GMYC model as implemented in Splits, code written by T. Ezard, T. Fujisawa and T. Barraclough in R v.3.0.2^48^ to compare the number of ESUs obtained from the single gene (COI: 540 bp; 145 taxa) and the two-gene (COI: 540 bp; 28S: 826 bp; 145 taxa) data sets. The ultrametric trees based on the COI and the combined data sets were obtained with Beast using a strict clock model without fossil calibrations and a Yule tree prior. Both data sets included all 120 unique haplotypes from Cape Verde samples and remaining Fissurellidae retrieved from GenBank (accession numbers in Table 1 Suplementary material). The analyses ran for 10,000,000 generations with sample frequency of 1000, after a burn-in of 1000,000.

We also used spedeSTEM^49^ to delimit the number of fissurellid species in Cape Verde by comparing the probability of models where putative evolutionary lineages are separate entities to the probability of models where putative lineages are collapsed into a single lineage using a maximum likelihood approach. SpedeSTEM takes as an input ultrametric trees and a user-supplied estimate of theta returning a table of models ranked according to their probability. Ultrametric trees based on the COI and 28S data sets were produced by Beast using the COI (540 bp; 145 taxa) and the 28S (826 bp; 145 taxa) data sets. The analyses ran for 10,000,000 generations with sample frequency of 1000, after a burn-in of 1000,000. We used Dnasp v.5^50^ to compute the average θ for the two loci.

### Dating analysis and diversification rates through time

To estimate the age of the most recent common ancestor of Cape Verde Fissurellidae we used Beast v.2.1.3^40^ that allows incorporation of fossil uncertainties. The data set used in this analysis (35 taxa; COI: 540 bp; 28S rRNA: 826 bp) included a single representative from each Cape Verde species inferred by ABGD and SpedeSTEM and the remaining Fissurellidae used in previous analyses. The calibration points used in this analysis are described in the Supplementary information.

We used BAMM (Bayesian analysis of macroevolutionary mixtures; www.bamm-project.org)^51^ to investigate heterogeneity in diversification rates in the Fissurellidae timetree obtained with Beast. This program uses MCMC simulations and reversible-jump sampling to estimate time-varying rates of speciation and extinction, and to find the optimal set of rate-shift configurations. Beast ultrametric tree was used for this analysis. We set four reversible jumping-MCMC running for 10 million generations sampled every 1000 generations and a burn-in of 10%. The function setBAMMpriors in R was used to choose more appropriate prior values. We used ESS (effective sample size) to assess the convergence of the runs and considered values above 200 as indicating convergence.

### Biogeographic analyses

We used the R package BioGeoBears (https://cran.r-project.org/web/packages/BioGeoBEARS/index.html)^52^,^53^ to estimate the ancestral ranges of Fissurellidae. Full description of the method is available on Supplementary information S1. We defined 13 geographical areas: (1) Sal; (2) Boavista; (3) Maio; (4) Santo Antão; (5) Santiago; (6) Ilhéu Raso; (7) Santa Luzia; (8) São Vicente; (9) São Nicolau; (10) Mediterranean; (11) western Atlantic; (12) Pacific, and (13) Africa. The maximum number of areas that any species may occur was set to five because it is the maximum number of areas where the species may occur.

### Shore substrate composition

To characterize shore substrate composition along the studied area, we prepared a 0.01° arc-degree (approx. 1.6 km at 16° N) mesh using R (R Development Core Team, 2014). The mesh grid was then imported to Google Earth, and all tiles covering both ocean and land were assigned a substrate type by means of visual inspection. Substrate types used were “rock”, “sand” or “both”. Lastly, the substrate type layers produced in Google Earth were rasterised using the R package raster^54^, and the amount of each substrate was quantified.

### Dispersal potential of keyhole limpets

Lagrangian Particle Simulations (LPS)^55^ were performed to estimate the dispersal potential of keyhole limpets throughout the Cape Verde archipelago. The simulations followed the standardized methods of Assis *et al*.^56^ and Klein *et al*.^57^ and used data assembled from the Hybrid Coordinate Ocean Model (HYCOM), a high-resolution product delivering ocean current fields on a daily basis. The region of simulation comprised ~1200 km of coastline, which was gridded to a spatial resolution of 0.005° (approx. 500 m). Individual particles simulating pelagic states were released every 12 hours from the centroids of each coastal cell with rocky reefs (shore substrate composite) from August to December for a 10-year period. We selected this period because natural spawning (e.g. in *Fissurella nigra*) occurs predominantly between October and December although organisms kept in laboratory spawned between August and November^17^. Considering that we have no information regarding Cape Verde species we included the higher possible interval (August - December) in all simulations. Passive particles were allowed to drift for 4 and 30 days until ending up on shore. The simulations were run for 4 and 30 days because the exact PLD is unknown for Cape Verde Fissurellidae. The asymmetric degree of connectivity between islands was inferred with network analysis, using percolation theory and the leading eigenvector community algorithm. Full description of the method is available on the Supplementary information.

## Additional Information

**How to cite this article**: Cunha, R. L. *et al*. Drivers of Cape Verde archipelagic endemism in keyhole limpets. *Sci. Rep.* **7**, 41817; doi: 10.1038/srep41817 (2017).

**Publisher's note:** Springer Nature remains neutral with regard to jurisdictional claims in published maps and institutional affiliations.

## Supplementary Material

Supplementary Information

## Figures and Tables

**Table 1:** Maximum and average distances, probabilities and drifting time produced by the particles that effectively connected different coastal cells determined for the simulations running 4 and 30 days of passive dispersal.

Dispersal time	Distance (km)		Probability		Time (days)	
	Maximum	Mean (±SD)	Maximum	Mean (±SD)	Maximum	Mean (±SD)
4 days	349.3	75.3 ± 75.9	0.791	0.003 ± 0.017	4,0	1.29 ± 0.85
30 days	349.3	76.1 ± 75.9	0.791	0.003 ± 0.017	11.95	1.35 ± 1.02

**Table 2:** Mean connectivity matrix between pairs of islands produced with the simulation running 4 days of passive dispersal.

	Santiago	Maio	Boavista	Sal	Sao Nicolau	Ilheu Raso	Santa Luzia	Sao Vincente	Santo Antao
**Santiago**	—	3.40E-04	1.40E-04	4.20E-05	7.80E-04	1.80E-04	4.10E-04	9.70E-05	1.10E-04
**Maio**	3.50E-03	—	2.00E-05	2.00E-05	3.70E-04	9.00E-05	1.90E-04	5.80E-05	4.90E-05
**Boavista**	5.70E-04	3.40E-04	—	2.60E-04	6.90E-04	8.40E-05	2.20E-04	7.70E-05	1.10E-04
**Sal**	3.60E-04	1.60E-04	7.00E-04	—	1.50E-03	1.50E-04	5.00E-04	3,80E-04	2.90E-04
**Sao Nicolau**	6.20E-04	1.20E-04	1.90E-04	1.20E-04	—	1.80E-03	1.90E-03	7.40E-04	7.80E-04
**Ilheu Raso**	6.40E-05	6.40E-06	1.10E-05	1.20E-05	1.60E-04	—	6.40E-04	1.90E-04	2.30E-04
**Santa Luzia**	3.30E-05	4.90E-06	2.80E-05	2.50E-05	2.50E-04	1.80E-04	—	1.50E-03	8.40E-04
**Sao Vincente**	1.70E-05	4.10E-06	1.60E-05	1.60E-05	9.60E-05	1.00E-04	1.00E-03	—	2.20E-03
**Santo Antao**	4.90E-06	2.30E-06	2.30E-05	5.50E-06	2.00E-04	1.00E-04	7.00E-04	1.30E-03	—

**Table 3:** Mean connectivity matrix between pairs of islands produced with the simulation running 30 days of passive dispersal.

	Santiago	Maio	Boavista	Sal	Sao Nicolau	Ilheu Raso	Santa Luzia	Sao Vincente	Santo Antao
**Santiago**	—	3.50E-04	1.40E-04	4.30E-05	7.90E-04	1.80E-04	4.50E-04	9.90E-05	1.10E-04
**Maio**	4.70E-03	—	2.00E-05	2.00E-05	3.80E-04	9.70E-05	2.20E-04	5.90E-05	5.00E-05
**Boavista**	6.20E-04	3.60E-04	—	2.70E-04	7.80E-04	9.70E-05	2.70E-04	7.90E-05	1.10E-04
**Sal**	3.70E-04	1.70E-04	7.50E-04	—	2.10E-03	1.80E-04	8.10E-04	4.40E-04	3.10E-04
**Sao Nicolau**	6.60E-04	1.20E-04	2.00E-04	1.20E-04	—	1.90E-03	2.10E-03	7.70E-04	8.00E-04
**Ilheu Raso**	6.80E-05	6.40E-06	1.10E-05	1.20E-05	1.60E-04	—	6.80E-04	2.00E-04	2.40E-04
**Santa Luzia**	3.40E-05	4.90E-06	2.80E-05	2.50E-05	2.60E-04	1.90E-04	—	1.60E-03	8.70E-04
**Sao Vincente**	1.70E-05	4.10E-06	1.60E-05	1.60E-05	9.70E-05	1.00E-04	1.10E-03	—	2.30E-03
**Santo Antao**	4.90E-06	2.30E-06	2.30E-05	5.50E-06	2.00E-04	1.00E-04	7.40E-04	1.30E-03	—
