# Supplementary material for: Drivers of Cape Verde archipelagic endemism in keyhole limpets
Source: Sci Rep. 2017 Feb 2;7:41817. doi: 10.1038/srep41817 (PMC5288781; doi:10.1038/srep41817)
Supplement: Supplementary Information [file srep41817-s1.pdf]

Supplementary Information

Drivers of archipelagic endemism in keyhole limpets

REGINA L. CUNHA[1,*], JORGE ASSIS[1], CELINE MADEIRA[1], RUI SEABRA[2], FERNANDO P. LIMA[2], EVANDRO P. LOPES,[2,3], SUZANNE T. WILLIAMS[4] AND RITA CASTILHO[1]

[1]*Centre of Marine Sciences - CCMAR, Universidade do Algarve, Campus de Gambelas, 8005 - 139 Faro, Portugal*
[2]*CIBIO, Centro de Investigação em Biodiversidade e Recursos Genéticos, Universidade do Porto, Campus Agrário de Vairão 4485-661 Vairão, Portugal*
[3]*Universidade de Cabo Verde, Departamento de Engenharias e Ciências do Mar, CP 163, São Vicente, Cabo Verde*
[4]*Department of Life Sciences, The Natural History Museum, London SW7 5BD, United Kingdom*

*Corresponding author: Regina L. Cunha
CCMAR – Centre of Marine Sciences
Campus de Gambelas, Universidade do Algarve
8005-139 Faro, Portugal
Email: rcunha@ualg.pt

Supplementary Information – S1

Methods

Dating analysis and diversification rates. To estimate the age of the most recent common ancestor of Cape Verde Fissurellidae we used BEAST v.2.1.3 [1] that allows incorporation of fossil uncertainties. The data set used in this analysis (35 taxa; COI: 540 bp; 28S rRNA: 826 bp) included a single representative from each Cape Verde species inferred by ABGD and SpedeSTEM and the remaining Fissurellidae used in previous analyses. We used two calibration points, one ranging from 33.9 to 23 myr, which is based on the first occurrence of the genus *Fissurella* in the paleontological record reported from the Lower Oligocene [2], and the other on the geological age of the archipelago [27.4-25.6] myr [3-5]. The approach of using geological ages to calibrate

the tree “stems from the idea that a lineage that has diversified within an area and is endemic to that area most probably post-dates the origin of that area” [6]. Considering that Fissurellidae only occur in shallow water habitats and attached to intertidal rocks, we assumed that their appearance on the archipelago would only have been possible after the fully emergence of the islands. First calibration was modeled with a lognormal distribution, where 95% of the prior felt within the geological interval in which the fossil of the stem lineage of the genus *Fissurella* was discovered. Parameters were: mean in real space, M=2.52; standard deviation, S=1.0, and hard minimum bound, Offset=23.03. The second calibration based on the K-Ar geological dates for the origin of the Cape Verde islands ([27.4 - 25.6] myr) was also modeled with a lognormal distribution where the parameters were: M=0.42; S=1.0, and Offset=25.6.

Biogeographic analyses. We used the R package BIOGEOBEARS (https://cran.r-project.org/web/packages/BioGeoBEARS/index.html) [7,8] to estimate the ancestral ranges of Fissurellidae. BIOGEOBEARS calculates maximum likelihood estimates of the ancestral states (range inheritance scenarios) at speciation events by modeling transitions between discrete states (biogeographical ranges) along phylogenetic branches as a function of time. Available models include a likelihood version of DIVA (Dispersal - Vicariance Analysis [9]), Lagrange’s DEC model (Dispersal-Extinction-Cladogenesis [10]) and BayArea [11]. Additionally, it implements the parameter “+J” that describes founder-event speciation, which is fundamental in oceanic settings [8,12].

Dispersal potential of keyhole limpets. Lagrangian Particle Simulations (LPS) were performed to estimate the dispersal potential of keyhole limpets throughout the Cape

Verde archipelago. The simulations followed the standardized methods of Assis et al. [13] and Klein et al. [14] and used data assembled from the Hybrid Coordinate Ocean Model (HYCOM), a high-resolution product delivering ocean current fields on a daily basis. This is a model forced by heat flux, precipitation, wind stress and wind speed that is able to resolve meandering currents, eddies, filaments and fronts [15]. These are important mesoscale oceanographic processes required to accurately simulate passive dispersing larvae (e.g., [13,14,16]).

The region of simulation comprised ~1200km of coastline, which was gridded to a spatial resolution of 0.005° (approx. 500m). Individual particles simulating pelagic states were released every 12 hours from the centroids of each coastal cell with rocky reefs (refer to shore substrate composition) from August to November, and allowed to drift for 4 and 30 days until ending up on shore. The geographical position of each particle was determined every hour using the bilinear interpolation of HYCOM's ocean velocity fields. To account for the inter-annual variability in ocean flows, the simulations performed per season run individually per year for a period of 10 years (2003 to 2012).

The individual trajectories were used to infer the degree of connectivity between islands per year, by determining the number of particles released from island i that ended up on island j, divided by the total number of particles released throughout the study region in a given year. The mean connectivity between pairs of islands was then determined by averaging the annual matrices.

The asymmetrical degree of connectivity between islands was used in network analysis (i.e., graph theory) to better visualize stronger linkages. In this process, network percolation removed weak connectivity thresholds to a cutoff allowing to connect all islands (nodes) into a single network [17], while maximizing modularity, a

measure that quantifies the strength (or goodness of fit) of the backbone structure of a given network [18]. This approach avoids the generation of multiple disconnected networks and removes less informative and surplus connections. A membership was assigned to the nodes (clusters in a network) with the leading eigenvector algorithm [18], a widely used approach for detecting community structure in networks (e.g., [19]). This approach allowed delineating the subregions structured by oceanographic currents allowing higher connectedness. The significance of membership assignment was inferred by testing the proportion of 10^4 membership randomizations retrieving higher modularity values than observed. Dispersal simulations and network analysis were performed in R (R Development Core Team, 2014) using the packages: igraph [20], data.table [21], dismo [22], raster [23] and vegan [24].

References

1 Bouckaert, R. *et al.* BEAST 2: a software platform for Bayesian evolutionary analysis. *PLoS Comp Biol* **10**, e1003537 (2014).

2 McClean, J. H. Reinstatement of the Fissurellid subfamily Hemitominae, with the description of new genera, and proposed evolutionary lineage, based on morphological characters of shell and radula (Gastropoda: Vetigastropoda). *Malacologia* **54**, 407-427 (2011).

3 Mitchell-Thomé, R. C. *Geology of the Middle Atlantic Islands*. (Science Publishers, 1976).

4 Coello, J. *et al.* Evolution of the eastern volcanic ridge of the Canary Islands based on new K-Ar data. *J. Volcanol. Geoth. Res.* **53**, 251-274 (1992).

5 Grunau, H. R., Lehner, P., Cleintuar, M. R., Allenbach, P. & Bakker, G. in *Progress in Geodynamics*. (ed G. J. Borradaille) 90-118 (Royal Soc. Neth. Academy of Arts and Sciences, 1975).

6 Clark, J. R. *et al.* A comparative study in ancestral range reconstruction methods: retracing the uncertain histories of insular lineages. *Systematic Biology* **57**, 693-707 (2008).

7 Matzke, N. J. BioGeoBEARS: BioGeography with Bayesian (and Likelihood) Evolutionary Analysis in R Scripts. R package, version 0.2.1, published July 27, 2013 at: http://CRAN.R-project.org/package=BioGeoBEARS (2013).

8 Matzke, N. J. Probabilistic historical biogeography: new models for founder-event speciation, imperfect detection, and fossils allow improved accuracy and model-testing. *Front. Biogeogr.* **5** (2013).

9 Ronquist, F. Dispersal-vicariance analysis: A new approach to the quantification of historical biogeography. *Syst. Biol.* **46**, 195-203 (1997).

10 Ree, R. H. & Smith, S. A. Maximum likelihood inference of geographic range evolution by dispersal, local extinction, and cladogenesis. *Syst. Biol.* **57**, 4-14 (2008).
11 Landis, M. J., Matzke, N. J., Moore, B. R. & Huelsenbeck, J. P. Bayesian Analysis of Biogeography when the Number of Areas is Large. *Syst. Biol.* **62**, 789-804, doi:10.1093/sysbio/syt040 (2013).
12 Matzke, N. J. Model selection in historical biogeography reveals that founder-event speciation is a crucial process in island clades. *Syst. Biol.* **63**, 951-970 (2014).
13 Assis, J. *et al*. Oceanographic Conditions Limit the Spread of a Marine Invader along Southern African Shores. *PLoS ONE* **10**, e0128124 (2015).
14 Klein, M. *et al*. High interannual variability in connectivity and genetic pool of a temperate clingfish, matches oceanographic transport predictions. *Plos ONE* (2016).
15 Chassignet, E. P. *et al*. The HYCOM (HYbrid Coordinate Ocean Model) data assimilative system. *J Mar Syst* **65**, 60-83 (2007).
16 Lett, C. *et al*. A Lagrangian tool for modelling ichthyoplankton dynamics. *Environ Model Assess* **23**, 1210–1214 (2008).
17 Rozenfeld, A. F. *et al*. Network analysis identifies weak and strong links in a metapopulation system. *Proc Natl Acad Sci* **105**, 18824–18829 (2008).
18 Newman, M. E. J. Modularity and community structure in networks. *Proc Natl Acad Sci* **103**, 8577–8582 (2006).
19 Munwes, I. *et al*. The change in genetic diversity down the core-edge gradient in the eastern spadefoot toad (*Pelobates syriacus*). *Mol Ecol* **19**, 2675–2689. (2010).
20 Csardi, G. & Nepusz, T. The igraph software package for complex network research. *InterJournal, Complex Systems* **1695**, 1-9 (2006).
21 R package data-table for fast aggregation of large data (2015).
22 Hijmans, R. J., Phillips, S., Leathwick, J. & Elith, J. dismo: Species distribution modeling. *R package version 0.8-17* (2013).
23 raster: Geographic data analysis and modeling. R package version 2.1-25. http://CRAN.R-project.org/package=raster (2013).
24 Dixon, P. VEGAN, a package of R functions for community ecology. *Journal of Vegetation Science* **14**, 927-930 (2003).

Supplementary Information – S2A

Summary of likelihood results and values for the parameters d, e and j.

	LnL	numparams	d	e	j
DEC	-133.00	2	0.0054	0.0146	0.0000
DEC+J	-125.55	3	0.0034	1.00E-12	0.0267
DIVALIKE	-134.09	2	0.0062	0.011	0.0000
DIVALIKE+J	-128.75	3	0.0040	1.00E-12	0.0259
BAYAREALIKE	-149.74	2	0.0073	0.1053	0.0000
BAYAREALIKE+J	-119.92	3	0.0023	0.0022	0.0351

Supplementary Information – S2B

Summary of likelihood results and statistics for model comparison.

	alt	null	LnLalt	LnLnull	DFalt	DFnull	DF	Dstatistic	pval	test	tail
**1**	DEC+J	DEC	-125.5	-133.0	3	2	1	14.91	0.0001	chi-squared	one-tailed
**2**	DIVALIKE+J	DIVALIKE	-128.8	-134.1	3	2	1	10.66	0.0011	chi-squared	one-tailed
**3**	BAYAREALIKE+J	BAYAREALIKE	-119.9	-149.7	3	2	1	59.65	1.10E-14	chi-squared	one-tailed

Supplementary Information - S3

Figure 1. Number of 0.01x0.01 degree tiles by substrate type

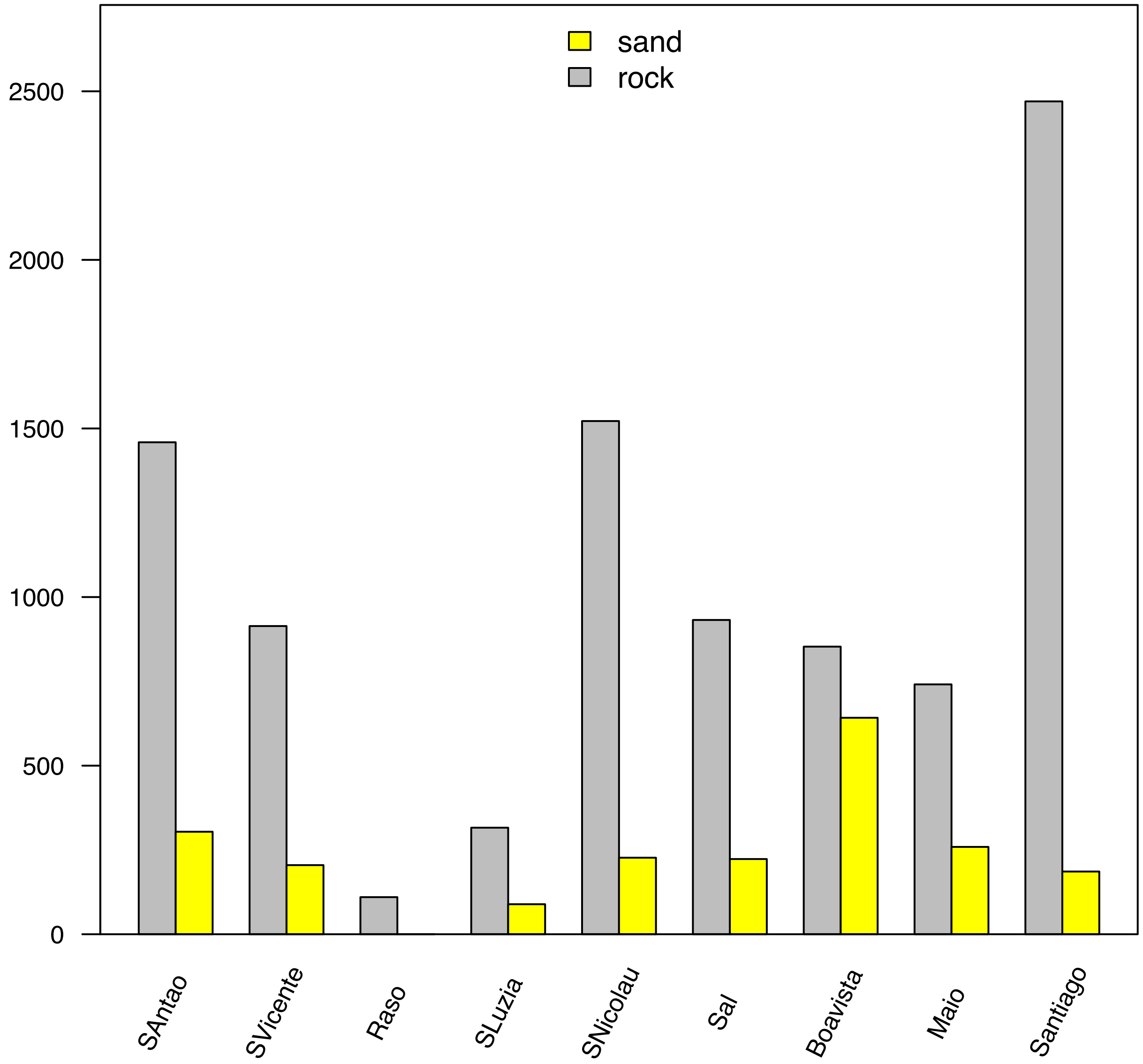

Supplementary Information - S4

Figure 2. Substrate type in each island (%)

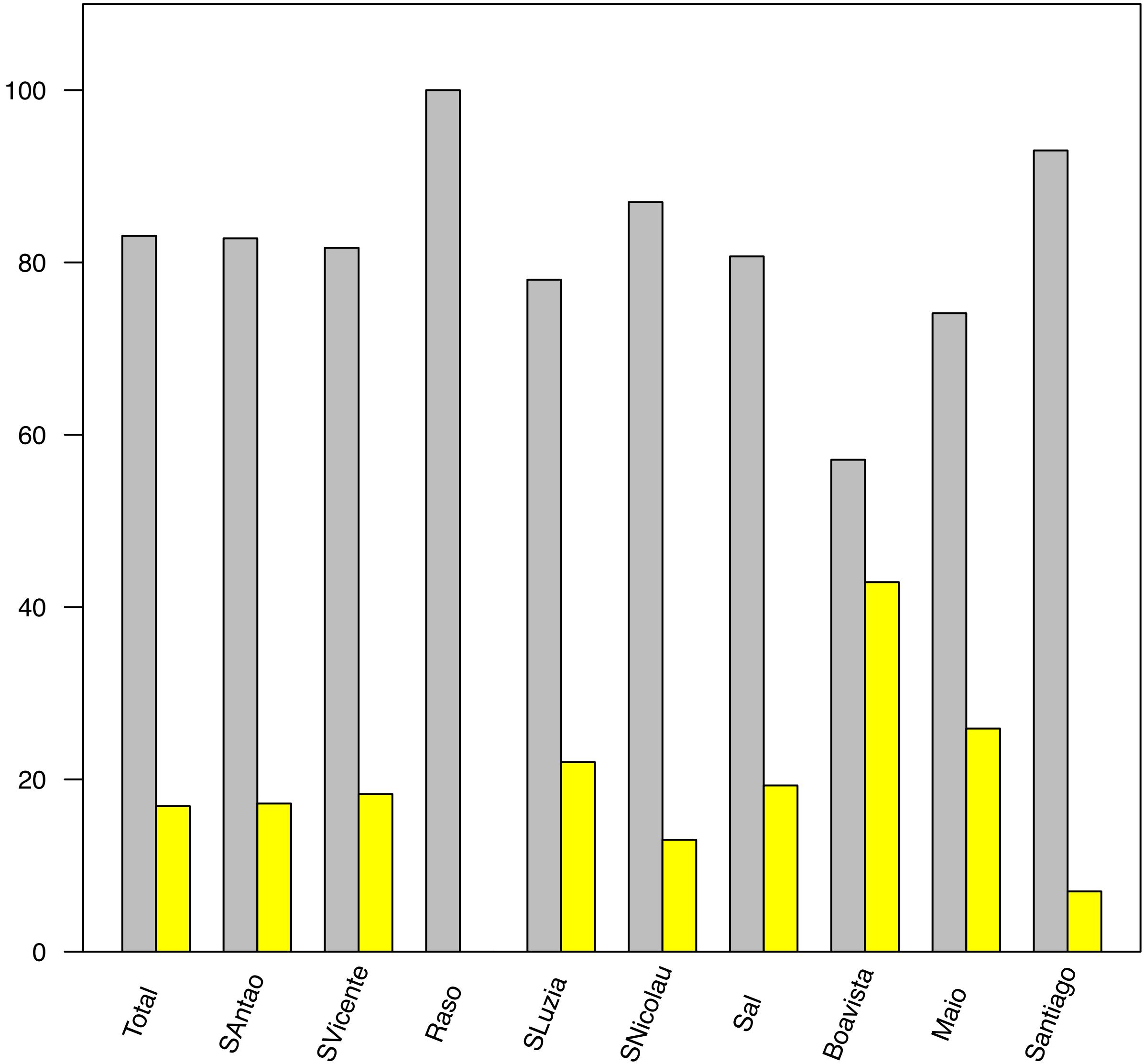

Table S1. Secimen codes, species names, geographic locations and Genbank accession numbers.

Code	Species	Geographic location	28S rRNA	COI
N/A	*Cranopsis cucullata*	California, West Atlantic	GQ160641	GQ160755
N/A	*Puncturella* sp.	Gulf of Mexico, West Atlantic	HM771473	HM771601
N/A	*Emarginula octaviana*	Spain, Mediterranean	HM771475	HM771603
N/A	*Emarginula variegata*	Queensland, Australia	HM771478	HM771605
N/A	*Emarginula foveolata fujitai*	Japan, Pacific Ocean	AM048697	AM049333
N/A	*Diodora graeca*	France, Mediterranean	HM771490	HM771613
N/A	*Diodora dysoni*	Bahamas, West Atlantic	FJ977669	FJ977754
N/A	*Diodora gibberula*	Spain, Mediterranean	HM771493	HM771614
N/A	*Diodora listeri*	Bermuda, West Atlantic	HM771495	HM771616
N/A	*Diodora lineata*	Sydney, Australia	HM771502	HM771621
N/A	*Diodora ticaonica*	Queensland, Australia	HM771503	HM771622
N/A	*Diodora singaporensis*	Queensland, Australia	HM771504	HM771623
N/A	*Diodora cayenensis*	North Carolina, USA	HM771499	HM771619
N/A	*Lucapina suffusa*	Panama, West Atlantic	FJ977673	FJ977758
N/A	*Fissurella* sp.	Cameroon, West Africa	HM771506	HM771624
N/A	*Fissurella nubecula*	Spain, Mediterranean	HM771507	HM771625
N/A	*Fissurella barbadensis*	Bahamas, West Atlantic	HM771511	HM771627
N/A	*Fissurella barbadensis*	Mexico, West Atlantic	HM771513	HM771629
N/A	*Fissurella* cf. *angusta*	Bahamas, West Atlantic	HM771514	HM771630
N/A	*Fissurella nodosa*	Bahamas, West Atlantic	FJ977671	FJ977756
N/A	*Macroschisma dilatatum*	Japan, Pacific Ocean	AM048698	AM049334
N/A	*Amblychilepas nigrita*	Sydney, Australia	HM771515	HM771631
N/A	*Sinezona confusa*	N/A	DQ279981	AF120631
N/A	*Lepetodrilus elevatus*	N/A	GQ160654	DQ093520
N/A	*Phorcus punctulatus*	N/A	JN686272	JN686360
**This study**			**28S rRNA**	**COI**
ANLU0021	Fissurellidae sp. 2	Luanda, Angola	KY204086	KY212763
ASHH0052	Fissurellidae sp. 1	Haga-Haga, South Africa	KY204087	KY212764
ASHH0053	Fissurellidae sp. 1	Haga-Haga, South Africa	KY204088	KY212765
ASHH0054	Fissurellidae sp. 1	Haga-Haga, South Africa	KY204089	KY212766
ASHH0056	Fissurellidae sp. 1	Haga-Haga, South Africa	KY204090	KY212767
BV000636	*Diodora philipiana*	Boavista, Cape Verde	KY204091	KY212768
BV000640	*Fissurella* cf. *salvatiana*	Boavista, Cape Verde	KY204092	KY212769
BV000647	*Fissurella bravensis*	Boavista, Cape Verde	KY204093	KY212770
BVDE0003	*Diodora philipiana*	Boavista, Cape Verde	KY204094	KY212771
BVGA0016	*Diodora philipiana*	Boavista, Cape Verde	KY204095	KY212772
BVGA0017	*Diodora philipiana*	Boavista, Cape Verde	KY204096	KY212773
BVSR0066	*Fissurella bravensis*	Boavista, Cape Verde	KY204097	KY212774
BVSR0070	*Fissurella bravensis*	Boavista, Cape Verde	KY204098	KY212775
BVSR0072	*Fissurella bravensis*	Boavista, Cape Verde	KY204099	KY212776
BVSR0073	*Fissurella bravensis*	Boavista, Cape Verde	KY204100	KY212777
BVSR0078	*Fissurella bravensis*	Boavista, Cape Verde	KY204101	KY212778
BVSR0080	*Fissurella bravensis*	Boavista, Cape Verde	KY204102	KY212779
BVSR0085	*Fissurella bravensis*	Boavista, Cape Verde	KY204103	KY212780
BVSR0190	*Fissurella bravensis*	Boavista, Cape Verde	KY204104	KY212781
BVSR0194	*Fissurella bravensis*	Boavista, Cape Verde	KY204105	KY212782
BVSR0197	*Fissurella bravensis*	Boavista, Cape Verde	KY204106	KY212783
BVSR0198	*Fissurella bravensis*	Boavista, Cape Verde	KY204107	KY212784
BVSR0199	*Fissurella bravensis*	Boavista, Cape Verde	KY204108	KY212785

ILRA0293	*Fissurella verna*	Ilhéu Raso, Cape Verde	KY204109	KY212786
ILRA0294	*Fissurella verna*	Ilhéu Raso, Cape Verde	KY204110	KY212787
ILRA0295	*Fissurella verna*	Ilhéu Raso, Cape Verde	KY204111	KY212788
ILRA0298	*Fissurella afra*	Ilhéu Raso, Cape Verde	KY204112	KY212789
ILRA0299	*Fissurella afra*	Ilhéu Raso, Cape Verde	KY204113	KY212790
ILRA0300	*Fissurella afra*	Ilhéu Raso, Cape Verde	KY204114	KY212791
ILRA0301	*Fissurella afra*	Ilhéu Raso, Cape Verde	KY204115	KY212792
ILRA0302	*Fissurella afra*	Ilhéu Raso, Cape Verde	KY204116	KY212793
ILRA0304	*Fissurella afra*	Ilhéu Raso, Cape Verde	KY204117	KY212794
ILRA0305	*Fissurella afra*	Ilhéu Raso, Cape Verde	KY204118	KY212795
MAPG0321	*Fissurella bravensis*	Maio, Cape Verde	KY204119	KY212796
MAPG0327	*Fissurella bravensis*	Maio, Cape Verde	KY204120	KY212797
MAPP0383	*Fissurella gaillardi*	Maio, Cape Verde	KY204121	KY212798
MAPP0386	*Fissurella gaillardi*	Maio, Cape Verde	KY204122	KY212799
MAPP0391	*Fissurella bravensis*	Maio, Cape Verde	KY204123	KY212800
MAPP0392	*Fissurella bravensis*	Maio, Cape Verde	KY204124	KY212801
MAPP0394	*Fissurella bravensis*	Maio, Cape Verde	KY204125	KY212802
MAPR0518	*Fissurella bravensis*	Maio, Cape Verde	KY204126	KY212803
MAPR0526	*Fissurella bravensis*	Maio, Cape Verde	KY204127	KY212804
MAPR0528	*Fissurella bravensis*	Maio, Cape Verde	KY204128	KY212805
MAPR0530	*Fissurella bravensis*	Maio, Cape Verde	KY204129	KY212806
MAPS0580	*Fissurella bravensis*	Maio, Cape Verde	KY204130	KY212807
SACU0591	*Fissurella verna*	Santo Antão, Cape Verde	KY204131	KY212808
SACU0592	*Fissurella afra*	Santo Antão, Cape Verde	KY204132	KY212809
SACU0593	*Fissurella afra*	Santo Antão, Cape Verde	KY204133	KY212810
SACU0597	*Fissurella afra*	Santo Antão, Cape Verde	KY204134	KY212811
SACU0603	*Fissurella afra*	Santo Antão, Cape Verde	KY204135	KY212812
SACU0605	*Fissurella afra*	Santo Antão, Cape Verde	KY204136	KY212813
SAPN0306	*Fissurella verna*	Santo Antão, Cape Verde	KY204137	KY212814
SAPN0313	*Fissurella verna*	Santo Antão, Cape Verde	KY204138	KY212815
SAPN0316	*Fissurella verna*	Santo Antão, Cape Verde	KY204139	KY212816
SAPS4853	*Fissurella afra*	Santo Antão, Cape Verde	KY204140	KY212817
SAPS4855	*Fissurella afra*	Santo Antão, Cape Verde	KY204141	KY212818
SAPS4857	*Fissurella afra*	Santo Antão, Cape Verde	KY204142	KY212819
SASI0279	*Fissurella afra*	Santo Antão, Cape Verde	KY204143	KY212820
SASI0284	*Fissurella afra*	Santo Antão, Cape Verde	KY204144	KY212821
SUAD0208	*Fissurella afra*	Santa Luzia, Cape Verde	KY204145	KY212822
SUAD0210	*Fissurella afra*	Santa Luzia, Cape Verde	KY204146	KY212823
SUAD0212	*Fissurella verna*	Santa Luzia, Cape Verde	KY204147	KY212824
SLBU0534	*Fissurella bravensis*	Sal, Cape Verde	KY204148	KY212825
SLBU0541	*Fissurella* sp. 2	Sal, Cape Verde	KY204149	KY212826
SLBU0542	*Fissurella bravensis*	Sal, Cape Verde	KY204150	KY212827
SLBU0543	*Fissurella bravensis*	Sal, Cape Verde	KY204151	KY212828
SLBU0545	*Fissurella bravensis*	Sal, Cape Verde	KY204152	KY212829
SLCA0481	*Fissurella bravensis*	Sal, Cape Verde	KY204153	KY212830
SLFR0548	*Fissurella bravensis*	Sal, Cape Verde	KY204154	KY212831
SLFR0549	*Fissurella bravensis*	Sal, Cape Verde	KY204155	KY212832
SLFR0551	*Fissurella bravensis*	Sal, Cape Verde	KY204156	KY212833
SLFR0557	*Fissurella bravensis*	Sal, Cape Verde	KY204157	KY212834
SLMU0247	*Fissurella bravensis*	Sal, Cape Verde	KY204158	KY212835
SLMU0249	*Fissurella bravensis*	Sal, Cape Verde	KY204159	KY212836
SLMU0252	*Fissurella bravensis*	Sal, Cape Verde	KY204160	KY212837
SLMU0254	*Fissurella bravensis*	Sal, Cape Verde	KY204161	KY212838
SLMU0256	*Fissurella bravensis*	Sal, Cape Verde	KY204162	KY212839

SLPL4792	*Fissurella* sp. 2	Sal, Cape Verde	KY204163	KY212840
SLPL4794	*Fissurella* sp. 1	Sal, Cape Verde	KY204164	KY212841
SLPL4795	*Fissurella* sp. 1	Sal, Cape Verde	KY204165	KY212842
SLPL4798	*Fissurella* sp. 1	Sal, Cape Verde	KY204166	KY212843
SLPL4805	*Fissurella bravensis*	Sal, Cape Verde	KY204167	KY212844
SLPL4806	*Fissurella bravensis*	Sal, Cape Verde	KY204168	KY212845
SLPL4809	*Fissurella* sp. 1	Sal, Cape Verde	KY204169	KY212846
SLPL4813	*Fissurella* sp. 1	Sal, Cape Verde	KY204170	KY212847
SLPL4814	*Fissurella* sp. 1	Sal, Cape Verde	KY204171	KY212848
SNPB0412	*Fissurella* sp. 2	São Nicolau, Cape Verde	KY204172	KY212849
SNPB0417	*Fissurella* sp. 2	São Nicolau, Cape Verde	KY204173	KY212850
SNPB0419	*Fissurella afra*	São Nicolau, Cape Verde	KY204174	KY212851
SNPB0423	*Fissurella* sp. 2	São Nicolau, Cape Verde	KY204175	KY212852
SNPR0427	*Fissurella* sp. 2	São Nicolau, Cape Verde	KY204176	KY212853
SNPR0435	*Fissurella* sp. 2	São Nicolau, Cape Verde	KY204177	KY212854
SNPR0436	*Fissurella afra*	São Nicolau, Cape Verde	KY204178	KY212855
SNPR0437	*Fissurella afra*	São Nicolau, Cape Verde	KY204179	KY212856
SNTA0397	*Fissurella afra*	São Nicolau, Cape Verde	KY204180	KY212857
SNTA0399	*Fissurella afra*	São Nicolau, Cape Verde	KY204181	KY212858
SNTA0400	*Fissurella afra*	São Nicolau, Cape Verde	KY204182	KY212859
SNTA0408	*Fissurella afra*	São Nicolau, Cape Verde	KY204183	KY212860
STCV4825	*Fissurella bravensis*	Santiago, Cape Verde	KY204184	KY212861
STCV4830	*Fissurella bravensis*	Santiago, Cape Verde	KY204185	KY212862
STCV4832	*Fissurella bravensis*	Santiago, Cape Verde	KY204186	KY212863
STCV4835	*Fissurella bravensis*	Santiago, Cape Verde	KY204187	KY212864
STCV4846	*Fissurella bravensis*	Santiago, Cape Verde	KY204188	KY212865
STCV4847	*Fissurella bravensis*	Santiago, Cape Verde	KY204189	KY212866
SVBG0217	*Fissurella verna*	São Vicente, Cape Verde	KY204190	KY212867
SVBG0218	*Fissurella afra*	São Vicente, Cape Verde	KY204191	KY212868
SVBG0222	*Fissurella afra*	São Vicente, Cape Verde	KY204192	KY212869
SVBG0224	*Fissurella afra*	São Vicente, Cape Verde	KY204193	KY212870
SVBG0225	*Fissurella verna*	São Vicente, Cape Verde	KY204194	KY212871
SVPC0034	*Fissurella bravensis*	São Vicente, Cape Verde	KY204195	KY212872
SVPC0036	*Fissurella bravensis*	São Vicente, Cape Verde	KY204196	KY212873
SVPC0038	*Fissurella bravensis*	São Vicente, Cape Verde	KY204197	KY212874
SVPC0041	*Fissurella bravensis*	São Vicente, Cape Verde	KY204198	KY212875
SVPG0266	*Fissurella afra*	São Vicente, Cape Verde	KY204199	KY212876
SVPG0267	*Fissurella afra*	São Vicente, Cape Verde	KY204200	KY212877
SVPG0270	*Fissurella afra*	São Vicente, Cape Verde	KY204201	KY212878
SVPG0271	*Fissurella afra*	São Vicente, Cape Verde	KY204202	KY212879
SVPG0272	*Fissurella afra*	São Vicente, Cape Verde	KY204203	KY212880
SVSP0457	*Fissurella verna*	São Vicente, Cape Verde	KY204204	KY212881
SVSP0469	*Fissurella afra*	São Vicente, Cape Verde	KY204205	KY212882
